# Poor short-term glycemic control in patients with type 2 diabetes impairs the intestinal mucosal barrier: a prospective, single-center, observational study

**DOI:** 10.1186/s12902-019-0354-7

**Published:** 2019-03-08

**Authors:** Lijuan Shen, Li Ao, Haoben Xu, Junfeng Shi, Dali You, Xiuwen Yu, Weixin Xu, Jie Sun, Fei Wang

**Affiliations:** 1 Department of Clinical Laboratory, Jiading District Central Hospital Affiliated Shanghai University of Medicine and Health Sciences, No.1, Chengbei Rd, Jiading District, Shanghai, 201800 China; 2 Department of Endocrinology, Jiading District Central Hospital Affiliated Shanghai University of Medicine and Health Sciences, Shanghai, 201800 China; 3 Anting Town Community Healthcare Center of Jiading District, Shanghai, 201805 China; 4 Shanghai Key Laboratory for Molecular Imaging, Shanghai University of Medicine & Health Sciences, Shanghai, 201318 China; 5 Department of Critical Care Medicine, Jiading District Central Hospital Affiliated Shanghai University of Medicine & Health Sciences, No.1, Chengbei Rd, Jiading District, Shanghai, 201800 China

**Keywords:** Type 2 diabetes mellitus, Intestinal mucosal barrier, Endotoxin, Blood glucose volatility, Intestinal permeability

## Abstract

**Background:**

To determine the relation between daily glycemic fluturation and the intestinal mucosal barrier dysfunction in type 2 diabetes mellitus (T2DM).

**Methods:**

Totally 66 patients with T2DM were enrolled, 33 healthy volunteers were also recruited according to the enrolled patients’ gender and age in a ratio of 2: 1. Patients were bisected by the median of endotoxins level into low(< 12.31 μ/l, *n* = 33) and high(≥12.31 μ/l, n = 33) blood endotoxin groups. Clinical data and blood glucose fluctuations were compared between groups. Multivariate regression analysis was used to determine the independent factors affecting the intestinal mucosal barrier.

**Results:**

Serum endotoxin [12.1 (4.2~22.0) vs 3.2 (1.3~6.0), *P* < 0.001] and fasting blood glucose levels [9.8 ± 3.6 vs 5.4 ± 0.7, P < 0.001] were significantly higher in patients with T2DM than the control group. The standard deviation of blood glucose (SDBG) within 1 day [2.9 (2.0~3.3) vs. 2.1 (1.6~2.5), *P* = 0.012] and the largest amplitude of glycemic excursions (LAGE) [7.5 (5.4~8.9) vs. 5.9 (4.3~7.4), *P* = 0.034] were higher in the high endotoxin group than in the low endotoxin group. A multiple linear stepwise regression revealed a positive correlation between SDBG with endotoxin (standard partial regression coefficient = 0.255, *P* = 0.039).

**Conclusions:**

T2DM patients who incapable of maintaining stable blood glucose level are at a higher risk to associated with intestinal mucosal barrier injury.

## Background

Type 2 diabetes mellitus (T2DM) is a long-term metabolic disorder caused by both genetic and environmental factors. The defects in insulin secretion or function (or both) can cause disorder in carbohydrates, proteins, fats, electrolytes, and water metabolism [1–3]. It is clinically characterized by chronic persistent hyperglycemia and high volatility. T2DM patients also tend to be associated with a series of chronic complications, such as nerve, blood vessel and gastrointestinal tract defects including intestinal mucosal barrier damage, seriously affecting the life quality [4, 5]. According to the latest statistics from the National Vital Statistics System, diabetes is the seventh of the top ten causes of death in the United States in 2016 [6].

The intestine is an important organ that should not be omitted during the treatment of DM [7–9]. The intestinal mucosal barrier prevents the translocation of bacteria and endotoxins into blood and lymph circulatory systems under normal physiological conditions. Dysregulation of intestinal mucosal barrier function would increase the permeability to the intestinal pathogens and endotoxins, which cause infection or inflammation [10, 11]. Previous studies suggested that the low-grade inflammatory state of patients with T2DM was possibily related to intestinal endotoxin [12, 13]. On the other hand, disorder of intestinal microflora composition is associated with the intestinal mucosal barrier damages [14, 15], which leads to nonspecific inflammatory, and in turn aggravates insulin resistance and T2DM metabolism disorders through the NF-κB pathway and JNK signal transduction pathway [16–18]. Therefore, the intestinal mucosal barrier function of patients with T2DM should be carefully monitored.

Currently, levels of the serum D-lactic acid, diamine oxidase (DAO), and endotoxin, which reflect the permeability of intestinal mucosa and bacterial translocation, are used to determine the intestinal mucosal barrier function in clinical practice [19, 20]. For patients with T2DM, glycemic monitoring (including short- and long-term blood glucose level fluctuations, as well as average blood glucose levels) is substantial. Studies have shown that persistent hyperglycemia can lead to intestinal mucosal barrier damage [5], however, the relationship between fluctuation of blood glucose level and intestinal mucosal barrier damage remains unclear. Therefore, this study aimed to investigate the relationship between glycemic control and intestinal mucosal barrier dysfunction in patients with T2DM.

## Methods

### Subjects

We recruited 66 patients diagnosed with T2DM from September 2017 to June 2018. All patients included in the study met the diagnostic criteria of the 2018 AACE/ACE Consensus Statement: Comprehensive Management of T2DM [21]. And their eating pattern were controlled according to the suggestion of ‘Standards of Medical Care in Diabetes’ in 2014 [22]. The control group was composed of 33 healthy volunteers who were recruited in a 2:1 ratio according to the patients’ sex and age. All subjects with digestive diseases, chronic malnutrition, malignant tumors, and intestinal infections which can inhibit the intestinal mucosal barrier function within 2 weeks were excluded.

The study protocol was approved by the Ethics Review Board of Jiading District Central Hospital (2017-ZD-03). All subjects were anonymized. All subjects signed informed consent form.

### Study methods

For all subjects included, the fasting venous blood sample was collected, the fasting blood glucose was examined, and the functionality of intestinal mucosal barrier of subjects were determined by the serum D-lactic acid, DAO, and endotoxin. Patients with T2DM were further divided into the low-value group (< 12.31u/l, *n* = 33) and high-value group (≥12.31u/l, n = 33) based on the median endotoxin levels. For the patients with T2DM, clinical characteristic were collected [DM duration, body mass index (BMI), history of hypertension, use of drugs (such as insulin and metformin etc.), smoking history, and family history of DM]. The abnormal fasting blood glucose, 2 h blood glucose level after breakfast, glycated hemoglobin (HbA1c), and glycated albumin (GA) of patients were also examined at the first day of enrollment. The daily blood glucose level (before breakfast (A), 2 h after breakfast (B), before lunch (C), 2 h after lunch (D), before dinner (E), 2 h after dinner (F), and before sleep (G)) was monitered by active blood glucose meter (Accu-Chek®, Germany) using the finger capillary blood samples. Short-term glycemic excursions, including the magnitude of postprandial glucose excursion (PPGE), the largest amplitude of glycemic excursions (LAGE), and the standard deviation of blood glucose (SDBG) within 1 day were calculated by:

PPGE = $$ \frac{\left(B-A\right)+\left(D-C\right)+\left(F-E\right)}{3} $$;

LAGE = D (maximum glycemic value) - A (minimum glycemic value);

SDBG =$$ \sqrt{\frac{\ {\left(A-X\right)}^2+{\left(B-X\right)}^2+{\left(C-X\right)}^2+{\left(D-X\right)}^2+{\left(E-X\right)}^2+{\left(F-X\right)}^2+{\left(G-X\right)}^2}{7-1}} $$;

X (glycemic average within 1 day) = $\frac{\left(A+B+C+D+E+F+G\right)}{7}$.

### Functionality of the intestinal mucosal barrier

The functionality of the intestinal mucosal barrier was determined using the DAO/lactic acid/bacterial endotoxin combined test kit (enzymatic method; Beijing Zhongsheng Jinyu Diagnostic Technology Co., Ltd.), supporting by the JY-DLT intestinal barrier function biochemical indicator analysis system. The experiments were undergone according to the protocols suggested by the manufacturer and conducted within 4 h after serum extraction.

### Statistical analysis

Statistical analysis was done using the SPSS 19.0 software (SPSS Inc., Chicago, IL, USA). Normally distributed data were expressed in mean ± standard deviations (SD) and compared using Student’s *t* test; otherwise was indicated as Median (Q1~Q3) and compared using the non-parametric Mann-Whitney test. Numerical data were expressed in frequency and compared using χ2 test. Multiple logistic regression was conducted to identify the factors that influenced the functionality of intestinal mucosal barrier. *P* < 0.05 was considered statistically significant.

## Results

### Baseline characteristics

The serum endotoxin [12.1(4.2~22.0) vs 3.2(1.3~6.0), *P* < 0.001] and FPG [(9.8 ± 3.6) vs (5.4 ± 0.7), P < 0.001] in patients with T2DM were significantly higher than those in the control group. No significant difference was observed in DAO and D-lactic acid (Table 1).

**Table 1:** Background characteristics of particippants

	Control groups (n = 33)	T2DM (*n* = 66)	Statistics	P
Male [n(%)]	19 (57.6)	38 (57.6)	*–*	1.000
Age [(Mean ± SD), years]	61.2 ± 9.6	60.9 ± 11.8	t = 0.121	0.904
BMI [(Mean ± SD), Kg/M^2^]	26.5 ± 5.8	25.1 ± 3.9	t = 1.417	0.160
Smoking [n(%)]	13 (39.4)	16 (24.2)	*x*^2^ = 2.438	0.118
Hypertension [n(%)]	16 (48.5)	40 (60.6)	*x*^2^ = 1.316	0.251
Diabetes history [(Mean ± SD), years]	NA	6.0 (1–14)	*–*	–
Diabetic complication [n(%)]	NA	31 (46.9)	*–*	–
Medications [n(%)]				
Insulin	NA	21 (31.8)	–	–
Metformin	NA	32 (48.5)	–	–
α-glucosidase inhibitor	NA	29 (43.9)	–	–
sulfonylureas	NA	9 (13.6)	–	–
glinide	NA	5 (7.6)	–	–
DPP-4 inhibitor	NA	1 (1.5)	–	–
Laboratory data				
HbAlc [M(Q1~Q3), %]	5.2 (4.9~5.6)	9.3 (7.4~11.0)	z = −7.340	< 0.001
GA [M(Q1~Q3), %]	14.1 (13.3~14.8)	23.5 (19.4~28.5)	z = −6.751	< 0.001
Creatinine [M(Q1~Q3), μmol/L]	76 (64.5~83)	65 (54.5~80)	z = −1.620	0.105
GFR [M(Q1~Q3), ml/min]	90.3 (83.7~98.8)	94.9 (78.1~104.1)	z = − 0.672	0.502
hs-CRP [M(Q1~Q3), ng/L]	3 (1.5~6.7)	3.5 (1.2~18.9)	z = −0.801	0.423
Index of the intestinal mucosal barrier				
DAO [M(Q1~Q3), u/l]	6.7 (5.4~9.3)	5.9 (4.1~9.6)	*z* = −1.413	0.158
D-lactic acid [M(Q1~Q3), mg/l]	49.0 (43.1~53.3)	47.0 (37.2~54.8)	*z =* −0.572	0.567
Endotoxins [M(Q1~Q3), μ/l]	3.2 (1.3~6.0)	12.1 (4.2~22.0)	*z* = −4.315	< 0.001
FPG[(Mean ± SD), mmol/l]	5.4 ± 0.7	9.8 ± 3.6	t = −9.070	< 0.001

*HbAlc* Glycosylated hemoglobin, *GA* glycated albumin, GFR Glomerular filtration rate, *DAO* diamine oxidase, *hs-CRP* high-sensitivity C-reactive protein, *FPG*:Fasting Plasma Glucose

### Clinical charateristics comparation between high- and low-endotoxin groups

The SDBG [2.9(2.0~3.3) vs 2.1(1.6~2.5), *P* = 0.012] and LAGE [7.5(5.4~8.9) vs 5.9(4.3~7.4), *P* = 0.034] in the high endotoxin group were higher than those in the low endotoxin group. No significant difference was observed in the other indicators (all *P* > 0.05) (Table 2).

**Table 2:** Between the two groups of clinical data and glucose control comparison

	Low groups (< 12.31u/l, n = 33)	High groups (12.31 ≥ u/l, n = 33)	Statistics	P
Male [n(%)]	16 (48.5)	22 (66.7)	*x*^2^ = 2.233	0.135
Age [(Mean ± SD), years]	62.3 ± 11.1	59.5 ± 12.5	t = 0.990	0.326
Hypertension [n(%)]	21 (63.6)	19 (57.6)	*x*^2^ = 0.254	0.614
Smoking [n(%)]	5 (15.2)	11 (33.3)	*x*^2^ = 2.970	0.085
Positive family history [n(%)]	7 (21.2)	7 (21.2)	–	1.000
BMI [(Mean ± SD), Kg/M^2^]	25.0 ± 4.4	25.2 ± 3.4	t = − 0.176	0.861
Diabetes history [(Mean ± SD), mouth]	9.0 (2~15.5)	5.0 (1.0~10.0)	*z* = −1.649	0.099
hs-CRP [M(Q1~Q3), mmol/l]	2.1 (0.5~6.6)	1.8 (0.5~6.7)	*z* = −0.250	0.802
Medications [n(%)]				
Insulin	11.0 (33.3)	10.0 (30.3)	*x*^2^ = 0.070	0.792
Metformin	17.0 (51.5)	15.0 (45.5)	*x*^2^ = 0.243	0.622
α-glucosidase inhibitor	12.0 (36.4)	17.0 (51.5)	*x*^2^ = 1.538	0.215
Sulfonylureas	2.0 (6.1)	7.0 (21.2)	–	0.149
Glinide	1.0 (3.0)	4.0 (12.1)	–	0.355
DPP-4 inhibitor	0 (0.0)	1 (3.0)	–	1.000
Glucose control data				
FPG [M(Q1~Q3), mmol/l]	8.6 (6.8~12.9)	8.8 (7.5~11.1)	*z* = − 0.023	0.982
2h PBG [M(Q1~Q3), mmol/l]	12.8 (10.6~18.1)	17.0 (15.2~20.8)	*z* = − 1.858	0.063
DGA [M(Q1~Q3), mmol/l]	11.0 (9.0~15.0)	11.9 (10.1~13.7)	*z* = − 0.301	0.763
HbAlc [M(Q1~Q3), %]	9.2 (8.2~10.5)	8.2 (8.2~9.4)	*z* = − 1.320	0.187
GA [M(Q1~Q3), %]	23.3 (19.3~27.2)	25.8 (20.0~36.3)	*z* = − 1.247	0.212
Glucose fluctuations of Short-term				
SDBG [M(Q1~Q3), mmol/l]	2.1 (1.6~2.5)	2.9 (2.0~3.3)	*z* = − 2.520	0.012
PPGE [M(Q1~Q3), mmol/l]	2.8 (1.8~3.1)	2.2 (1.9~3.8)	*z* = − 0.917	0.359
LAGE [M(Q1~Q3), mmol/l]	5.9 (4.3~7.4)	7.5 (5.4~8.9)	*z* = − 2.123	0.034
SDBG < 2.0 mmol/l [n(%)]	15.0 (45.5)	9.0 (27.3)	*x*^2^ = 2.357	0.125
PPGE < 2.2 mmol/l [n(%)]	15.0 (45.5)	11.0 (33.3)	*x*^2^ = 1.015	0.314
LAGE< 4.4 mmol/l [n(%)]	9.0 (27.3)	4.0 (12.1)	*x*^2^ = 2.395	0.122

*DGA* Daily glucose average,*FPG*:Fasting Plasma Glucose, *PBG*:Postprandial Blood Glucose, *SDBG* Standard Deviation Of Blood Glucose, *PPGE* Postprandial Glucose Excursion, *LAGE* Largest amplitude of glycemic excursions, *BMI* Body Mass Index, *HbAlc* Glycosylated hemoglobin, *GA* glycated albumin, *hs-CRP* high-sensitivity C-reactive protein

### Regression analysis

**Fig. 1:**
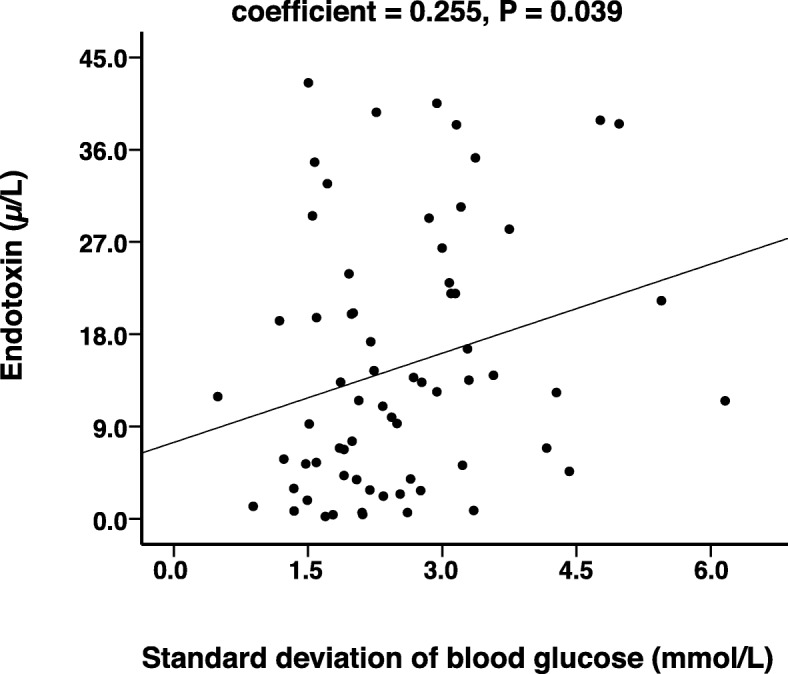
Scatter plot of the correlation between endotoxin and SDBG. SDBG: Standard Deviation Of Blood Glucose

Multivariable linear regression analysis showed that after adjusting for gender, age, and LAGE, SDBG was positively correlated with endotoxin independently (standard partial regression coefficient = 0.255, *P* = 0.039). The scatter-plot is shown in Fig. 1.

## Discussion

Previous reports exhibited that patients with T2DM are more susceptible to intestinal mucosal barrier dysfunction [23, 24], however factors remains to be investigated. In this study, T2DM patients with higher daily blood glucose fluctuation tended to have a higher serum endotoxin level, which indicates the function of the intestinal mucosal barrier was possibly compromised.

In this study patients with T2DM and high SDBG were associated with higher serum endotoxin level; meanwhile there was no significant change in serum DAO and D-lactic acid levels. Diamine oxidase (DAO) is an enzyme mainly produced in the small intestine involved in the histamine metabolism [25, 26]. Recent studies showed that a established first-line treatment for patients in T2DM, metformin, inhibits DAO activity [27]. Such inhabitation possibly compensate the increased DAO due to the dysfunction of intestinal mucosal barrier. D-lactate is a bydroxycarboxylic acid produced by bacterial fermentation [28, 29]. Studies revealed that the gut microbiome composition is altered for T2DM patients treated with metformin [30], in which possibly explain the inconsistent result in serum D-lactate and endotoxin level.

Endotoxins, also known as Lipopolysaccharides (LPS), are large molecules found in the outer membrane of Gram-negative bacteria. The increase in LPS is associated with bacterial translocation due to the impairment of intestinal epithelial cell [31]. The increase in endotoxins of T2DM patients are probably result from the change in gut microbial composition, epithelial cell impairment, responses to inflammatory mediators or secondary action of endotoxins [31–34]. A resent study on mice suggested that fluctuant hyperglycemia had more potential to cause oxidative stress and inflammation, and eventually endothelial dysfunction [35]. In this study, although the amplitude of blood glucose fluctuation of patients are much less than that of mouse model (2.1/2.9 mmol/l vs ~ 15 mmol/l), the SDBG is still an independent factor positively correlated to the serum LPS level. In addition, blood glucose fluctuations are associated with ketoacidosis in patients with DM, and also an independent risk factors for the progression of atherosclerosis [4, 36]. These pevious reports, combined with this study, further indicate the importance of blood glucose stabilization to health.

Previous studies revealed that T2DM patients are more probably associated with higher LPS, in which long-term hyperglycemia in these patients is one of the causes of intestinal mucosal barrier damage [37]. The compromised mucosal barrier could be a potential cause of the large blood glucose fluctuartion. In this study, however, no sign of inflammatory damage of intestinal mucosa was observed as the CRP level of patients are at normal level.

Multivariable linear regression analysis revealed that the amplitude of blood glucose fluctuations was an independent risk factor of increased intestinal permeability. The blood glucose fluctuation compromise the intestinal permeability in several possible ways: (1) Increased blood glucose fluctuations affect the integrity of the intestinal epithelial cells and the contact structure between cells [38]. (2) Abnormal metabolic pathways caused by fluctuations in blood glucose lead to increased inflammatory factors and stimulate intestinal mucosa [38]. (3) Function of liver is compromised due to the flucturation, thereby preventing timely and effective removal of endotoxins.

The positively correlation between intestinal mucosal barrier damage and SDBG indicates that clinicans/ T2DM patients should carefully maintain stable blood glucose level. Subsequent studies should be done to confirm our findings by increasing the sample size. Although DAO, D-lactic acid, and endotoxin can be used as indicators of the intestinal mucosal barrier status, the impairment mechanism of intestinal mucosal barrier remains to be clearly elucidated. Factors such as intestinal microenvironment affected by medication and immunity of patients should be further investigated.

## Conclusions

In summary, impairment of the intestinal mucosal barrier function, characterized by impaired intestinal permeability, may occur in patients with T2DM with large SDBG. Clinical attention should be focused on monitoring and controlling blood glucose fluctuations in patients with T2DM.

## Abbreviations

BMI: Body Mass Index
DAO: Diamine oxidase
DGA: Daily glucose average
FPG: Fasting Plasma Glucose
GA: glycated albumin
HbA1c: Glycosylated hemoglobin
hs-CRP: High-sensitivity C-reactive protein
LAGE: Largest amplitude of glycemic excursions
PBG: Postprandial Blood Glucose
PPGE: Postprandial glucose excursion
SDBG: Standard Deviation Of Blood Glucose
T2DM: Type 2 diabetes mellitus

## References

[1] Yang W, Lu J, Weng J (2010). Prevalence of diabetes among men and women in China. N Engl J Med.

[2] Malo MS (2015). A high level of intestinal alkaline phosphatase is protective against type 2 diabetes mellitus irrespective of obesity. EBioMedicine.

[3] Sato J, Kanazawa A, Ikeda F (2014). Gut dysbiosis and detection of "live gut bacteria" in blood of Japanese patients with type 2 diabetes. Diabetes Care.

[4] Zhao H, Li Z, Tian G (2013). Effects of traditional Chinese medicine on rats with type II diabetes induced by high-fat diet and streptozotocin: a urine metabonomic study. Afr Health Sci.

[5] Thaiss CA, Levy M, Grosheva I, et al. Hyperglycemia drives intestinal barrier dysfunction and risk for enteric infection. Science. 2018;359(6382):1376–83. 10.1126/science.aar3318 29519916

[6] Heron M (2018). Deaths: Leading Causes for 2016. Natl Vital Stat Rep.

[7] Hayes MT, Foo J, Besic V (2011). Is intestinal gluconeogenesis a key factor in the early changes in glucose homeostasis following gastric bypass?. Obes Surg.

[8] Li B1, Zhou X, Wu J (2013). From gut changes to type 2 diabetes remission after gastric bypass surgeries. Front Med.

[9] Koliaki C, Liatis S, le Roux CW (2017). The role of bariatric surgery to treat diabetes: current challenges and perspectives. BMC Endocr Disord.

[10] Scaldaferri F, Pizzoferrato M, Gerardi V (2012). The gut barrier: new acquisitions and therapeutic approaches. J Clin Gastroenterol.

[11] Yang X, Gao XC, Liu J (2017). Effect of EPEC endotoxin and bifidobacteria on intestinal barrier function through modulation of toll-like receptor 2 and toll-like receptor 4 expression in intestinal epithelial cell-18. World J Gastroenterol.

[12] Al-Attas OS, Al-Daghri NM, Al-Rubeaan K (2009). Changes in endotoxin levels in T2DM subjects on anti-diabetic therapies. Cardiovasc Diabetol.

[13] Creely SJ, McTernan PG, Kusminski CM (2007). Lipopolysaccharide activates an innate immune system response in human adipose tissue in obesity and type 2 diabetes. Am J Physiol Endocrinol Metab.

[14] Lozupone CA, Stombaugh JI, Gordon JI (2012). Diversity, stability and resilience of the human gut microbiota. Nature..

[15] Genton L, Cani PD, Schrenzel J (2015). Alterations of gut barrier and gut microbiota in food restriction, food deprivation and protein-energy wasting. Clinl Nutr..

[16] Cyphert TJ, Morris RT, House LM (2015). NF-κB-dependent airway inflammation triggers systemic insulin resistance. Am J Physiol Regul Integr Comp Physiol.

[17] Kaĭdashev IP (2012). NF-kB activation as a molecular basis of pathological process by metabolic syndrome. Fiziol Zh.

[18] Ferdaoussi M, Abdelli S, Yang JY (2008). Exendin-4 protects beta-cells from interleukin-1 beta-induced apoptosis by interfering with the c-Jun NH2-terminal kinase pathway. Diabetes.

[19] Xu J, Liu Z, Zhan W (2018). Recombinant TsP53 modulates intestinal epithelial barrier integrity via upregulation of ZO-1 in LPS-induced septic mice. Mol Med Rep.

[20] Gao X, Miao R, Tao Y (2018). Effect of montmorillonite powder on intestinal mucosal barrier in children with abdominal Henoch-Schonlein purpura: a randomized controlled study. Medicine (Baltimore).

[21] Garber AJ, Abrahamson MJ, Barzilay JI (2018). Consensus statement by the American Association of Clinical Endocrinologists and American College of endocrinology on the comprehensive type 2 diabetes management algorithm-2018 executive summary. Endocr Pract.

[22] American Diabetes Association. Standards of medical care in diabetes--2014. Diabetes Care. 2014;37(Suppl 1):S14–80. 10.2337/dc14-S014 24357209

[23] Zhong H, Yuan Y, Xie W, et al. Type 2 Diabetes Mellitus Is Associated with More Serious Small Intestinal Mucosal Injuries. PLoS ONE. 11(9):e0162354. 10.1371/journal.pone.0162354 PMC5012602 27598308

[24] Horton F, Wright J, Smith L (2014). Short Report: Pathophysiology Increased intestinal permeability to oral chromium (51Cr)-EDTA in human Type 2 diabetes. Diabet Med.

[25] Zhao L, Luo L, Jia W (2014). Serum diamine oxidase as a hemorrhagic shock biomarker in a rabbit model. PLoS One.

[26] Bragg LE, Thompson JS, West WW (1991). Intestinal diamine oxidase levels reflect ischemic injury. J Surg Res.

[27] Yee SW, Lin L, Merski M (2015). Prediction and validation of enzyme and transporter off-targets for metformin. J Pharmacokinet Pharmacodyn.

[28] Demircan M, Cetin S, Uguralp S (2004). Plasma D-lactic acid level: a useful marker to distinguish perforated from acute simple appendicitis. Asian J Surg.

[29] Sun XQ, Fu XB, Zhang R (2001). Relationship between plasma D(−)-lactate and intestinal damage after severe injuries in rats. World J Gastroenterol.

[30] McCreight LJ, Bailey CJ, Pearson ER (2016). Metformin and the gastrointestinal tract. Diabetologia..

[31] Cavaillon JM (2018). Exotoxins and endotoxins: inducers of inflammatory cytokines. Toxicon..

[32] Wu X, Ma C, Han L (2010). Molecular characterisation of the faecal microbiota in patients with type II diabetes. Curr Microbiol.

[33] Cani PD, Amar J, Iglesias MA (2007). Metabolic endotoxemia initiates obesity and insulin resistance. Diabetes.

[34] Al-Obaide MAI, Singh R, Datta P, et al. Gut Microbiota-Dependent Trimethylamine-N-oxide and Serum Biomarkers in Patients with T2DM and Advanced CKD. J Clin Med. 2017;6(9). 10.3390/jcm6090086 PMC5615279 28925931

[35] Wu N, Shen H, Liu H (2016). Acute blood glucose fluctuation enhances rat aorta endothelial cell apoptosis, oxidative stress and pro-inflammatory cytokine expression in vivo. Cardiovasc Diabetol.

[36] Martín-Timón I, Sevillano-Collantes C, Segura-Galindo A (2014). Type 2 diabetes and cardiovascular disease: have all risk factors the same strength?. World J Diabetes.

[37] Jayashree B, Bibin YS, Prabhu D (2014). Increased circulatory levels of lipopolysaccharide (LPS) and zonulin signify novel biomarkers of proinflammation in patients with type 2 diabetes. Mol Cell Biochem.

[38] Oliveira RB, Canuto LP, Collares-Buzato CB (2019). Intestinal luminal content from high-fat-fed prediabetic mice changes epithelial barrier function in vitro. Life Sci.

